# Alkaline Phosphatase: The Next Independent Predictor of the Poor 90-Day Outcome in Alcoholic Hepatitis

**DOI:** 10.1155/2013/614081

**Published:** 2013-09-17

**Authors:** Beata Kasztelan-Szczerbinska, Maria Slomka, Krzysztof Celinski, Mariusz Szczerbinski

**Affiliations:** Department of Gastroenterology with Endoscopy Unit, Medical University of Lublin, 8 Jaczewski Street, 20-954 Lublin, Poland

## Abstract

*Aim*. Determination of risk factors relevant to 90-day prognosis in AH. Comparison of the conventional prognostic models such as Maddrey's modified discriminant function (mDF) and Child-Pugh-Turcotte (CPT) score with newer ones: the Glasgow Alcoholic Hepatitis Score (GAHS); Age, Bilirubin, INR, Creatinine (ABIC) score, Model for End-Stage Liver Disease (MELD), and MELD-Na in the death prediction. *Patients and Methods*. The clinical and laboratory variables obtained at admission were assessed. The mDF, CPT, GAHS, ABIC, MELD, and MELD-Na scores' different areas under the curve (AUCs) and the best threshold values were compared. Logistic regression was used to assess predictors of the 90-day outcome. *Results*. One hundred sixteen pts fulfilled the inclusion criteria. Twenty (17.4%) pts died and one underwent orthotopic liver transplantation (OLT) within 90 days of follow-up. No statistically significant differences in the models‘ performances were found. Multivariate logistic regression identified CPT score, alkaline phosphatase (AP) level higher than 1.5 times the upper limit of normal (ULN), and corticosteroids (CS) nonresponse as independent predictors of mortality. *Conclusions*. The CPT score, AP > 1.5 ULN, and the CS nonresponse had an independent impact on the 90-day survival in AH. Accuracy of all studied scoring systems was comparable.

## 1. Introduction

Alcohol remains the main cause of liver disease in Europe. According to the 2011 World Health Organization (WHO) Global Status Report on Alcohol and Health, many eastern European countries, including Poland, have the highest consumption, risky patterns of drinking, and high levels of alcohol-related deaths and disabilities [1]. Polish adult per capita consumption (APC) in 2005 was equal to 13.3 liters of pure alcohol and much higher than the average alcohol consumption worldwide (5.1 liters of pure alcohol APC). A remarkable increase in the frequency of alcohol consumption and consequently in alcoholic liver disease (ALD) has been demonstrated mostly in Eastern Europe. The prevalence of the disorder, its high fatality rate, and lack of effective treatment keeps the disease in the focus of scientific investigations. Significant debate continues about patients' (pts) management and prognosis [2, 3].

Several scoring systems were proposed for prognostic stratification of pts with alcoholic hepatitis (AH). The Maddrey's modified discriminant function (mDF) has been used most frequently. Values greater than 32 indicate severe disease and predict a 30-day mortality rate of approximately 50% individuals [4]. The Model for End-Stage Liver Disease (MELD) score and the Glasgow Alcoholic Hepatitis Score (GAHS) [5] have been compared with the mDF and the Child-Pugh-Turcotte score (CPT) in some populations [6–9]. There is also a new tool developed recently called the Age, Bilirubin, International Normalized Ratio and Creatinine (ABIC) score [10].

Accordingly, we designed the prospective study to evaluate the clinical profile and the 3-month prognosis of Polish pts with AH. The second aim of our study was to compare accuracy of the traditional prognostic models as mDF and CPT score, and the newer ones GAHS, ABIC, MELD, and MELD-Na in the death prediction. The study was conducted in the Department of Gastroenterology with Endoscopy Unit of Medical University in Lublin, Poland.

## 2. Materials and Methods

Adult pts with AH were prospectively included over a 2-year period and followed for 90 days. The Alcohol Use Disorders Identification Test (AUDIT) developed by the WHO was used for identification of risky drinkers. An AUDIT score of ≥8 for men up to age of 60, ≥4 for women, or men over age 60 was considered a positive screening test [11]. All data were collected on admission. Subjects were confirmed to be abstinent from alcohol for at least 24 hours prior to blood sampling. No one was treated with corticosteroids or pentoxifylline at the time of admission. Patients by protocol completed AUDIT, physical examination, and laboratory tests to compute mDF, CPT, GAHS, ABIC, MELD, and MELD-Na scores (calculators from http://www.mayoclinic.org/ and http://potts-uk.com/livercalculator.html were applied). The diagnosis of AH was based on clinical criteria: a detailed patient history, typical symptoms and physical findings, laboratory values (elevated serum aminotransferases activity, the AST/ALT ratio higher than 2, a minimum bilirubin cut off 4.5 mg/dL), and imaging studies, in the setting of excessive alcohol intake (i.e., alcohol consumption exceeding 40 g/d for male and 20 g/d for female pts for a minimum of 6 months) [2, 12]. Since the most consistent difference between patients with AH and the other stages of ALD is the degree of hyperbilirubinemia (the reported accuracy of a clinical diagnosis of AH based on a minimum level of bilirubin ranging 80 to 100 *μ*mol/L was about 96%) [13], we used the level of 4.5 mg/d to select our patient cohort. Apart from a minimum level of bilirubin, we did not use a specific cutoff for AST and ALT. Furthermore, positive AUDIT in addition to the amount of alcohol consumption was an inclusion criterion. Further laboratory tests were used to exclude any other etiologies of chronic liver injury (hepatitis C and hepatitis infection, autoimmune hepatitis, Wilson disease, and hemochromatosis). To assess the influence of cholestatic enzymes, we followed the definition of cholestasis according to the European Association for the Study of the Liver (EASL) suggestion and decided to evaluate alkaline phosphatase (AP) levels higher than 1.5 times the upper limit of normal (ULN) and gamma-glutamyl transpeptidase (GGT) levels >3x ULN [14]. Ultrasonography of the abdomen was performed to confirm the presence of ascites and to rule out other causes of cholestasis (e.g., choledochal cyst, gallstones). Tests for antimitochondrial antibodies (AMA) to exclude the diagnosis of primary biliary cirrhosis (PBC) were done and drugs hepatotoxicity was ruled out. Liver biopsy was required in selected cases, when the diagnosis was unclear. Pts with any other severe associated diseases, that is, uncontrolled diabetes, heart failure, pulmonary insufficiency, or malignancy, at the time of inclusion were excluded. Only those individuals for whom all the required laboratory data were available at admission were included. Patients with severe AH manifested by an mDF ≥ 32, hepatic encephalopathy (HE), or both, received prednisone 40 mg daily. The response to corticosteroids (CS) was determined after 6–9 days of treatment (defined as bilirubin level lower than that on the first day of treatment) and if positive the treatment was continued for 4 weeks. After 28 days of initial therapy, the dose of prednisone was tapered by 5 mg per week and then stopped.

All patients included in the trial were inpatients at the starting point of the study. They generally were discharged from our department once alcohol withdrawal symptoms have disappeared, liver function has begun to improve, and complications of liver failure (i.e., jaundice, encephalopathy, coagulopathy, etc.) have resolved. Subsequent follow-up visits during next 90 days were set at least once a month (generally every 2 weeks) in the liver clinic or during any hospital admissions in the previously mentioned period if required. Four of nonsurvivors were admitted again after their condition worsened and they died in the hospital. The majority of nonsurvivors (17 of 21) were treated in our department without any hospital discharge.

The study protocol conforms to the ethical guidelines of the 1975 Declaration of Helsinki (6th revision, 2008) as reflected in a priori approval by the institutional review board of Medical University of Lublin.

## 3. Statistical Analysis

Statistical analysis was performed using the Statistica 10 software package (StatSoft, Poland). The distribution of the data in the groups was preliminarily evaluated by Kolmogorov and Smirnov test. Continuous variables were described both as mean with standard deviation and median with interquartile range and compared using Student's *t*-test or Mann-Whitney *U*-test, as appropriate, depending on their normality test. Differences between categorical variables were assessed by Fisher's exact test or the *χ* ^2^ test with Yates correction for continuity, when necessary. Univariate logistic regression was used to screen the variables reported as significantly different in nonsurvivors group for association with 3 month mortality. Variables that occurred statistically significant were further checked as potentially independent predictors of the poor 3-month outcome (logistic regression). The receiver operating curves (ROC) for all scores were constructed to assess different areas under the curve (AUCs) and the best threshold values for predicting 3-month outcome. The method of DeLong et al. (1988) for the calculation of the Standard Error of the AUC and of the difference between AUCs was used. Binomial exact Confidence Interval for the AUCs was calculated. The Youden index and its associated cut-off point was estimated for each model.

A two-sided *P* value of less than 0.05 was considered to be associated with statistical significance.

## 4. Results

### 4.1. Study Population and Their Clinical Profile

One hundred sixteen pts fulfilled inclusion criteria. Of the 116 pts with AH, 20 (17.4%) died from complications of liver failure within 3 months and one female underwent orthotopic liver transplantation (OLT). The patient who underwent OLT was included in the analysis by combining death and OLT as hard outcome. The survey population included 72 males (62.1%) and 44 females (37.9%). The mean age was 49.4 ± 10.7 (range from 28 to 69).

Ascites was present in 74 of 116 (63.8%) subjects and HE in 29 of 116 (25.0%).

Baseline characteristics of the study group are shown in Tables 1 and 2.

Overall survival at 30 and 90 days following the index admission was 99 of 116 (85.3%) and 95 of 116 (81.9%), respectively. All 21 (100%) nonsurvivors presented with ascites and encephalopathy was diagnosed in 11 of them (55.0%). Notably, the absence of both ascites and encephalopathy was associated with 100% survival at 3 months.

Corticosteroids (CS) were administered to 41 pts: 27 of them presented with mDF ≥ 32 and 14 with hepatic encephalopathy; both conditions were present in 15 of these pts.

The positive response to CS after 6–9 days of treatment was observed in 29 (70.7%) and the absence of response in 12 (29.3%) pts. There was statistically significant difference in the 90-day mortality between CS responders and nonresponders (9 out of 29 versus 10 out of 12, resp., *P* = 0.005). Univariate analysis revealed total bilirubin (*P* = 0.002), serum albumin (*P* = 0.000), INR (*P* = 0.000), serum sodium below 135 mEq/L (*P* = 0.005), AP > 1.5 ULN (*P* = 0.014), white blood cells count (WBC) (*P* = 0.03), and all scoring models as predictors of 3-month mortality. Hepatic encephalopathy (HE) (*P* = 0.005) and the CS nonresponse (*P* = 0.005) also predicted poor outcome.

The multivariate analysis included all the significant variables (NA ≤ 135 mEq/L, AP > 1.5 ULN, WBC, HE, and CS nonresponse) confirmed in the univariate analysis apart from the parameters which were calculated as a part of the scoring models. To avoid collinearity, ABIC, GAHS, MELD, MELD-Na, CPT, and mDF scores were incorporated and assessed in the separate statistical models. The CPT score, AP > 1.5 ULN and CS nonresponse were identified as independent predictors of the poor short-term survival. The other models and variables lost their significance when adjusted for AP > 1.5 ULN and the CS nonresponse. Results of logistic regression are summarized in Tables 3 and 4.

### 4.2. Scoring Systems Validation

Baseline values of six models used for prognosis assessment are provided in Table 2. All studied scoring systems were significant predictors of 90-day mortality. Results of univariate logistic regression are shown in Table 3. The CPT and MELD-NA had the best accuracy as demonstrated by their AUCs (although not statistically better than the others). The highest specificity was confirmed for the two newest models (GASH and ABIC), but CPT, MELD, and MELD-Na were better regarding a higher sensitivity. The most compelling argument for poor prognosis was ABIC greater than 8.46 (+ LR  5.94) and against it MELD-Na equal to or lower than 21 (− LR  0.22).

**Figure 1:**
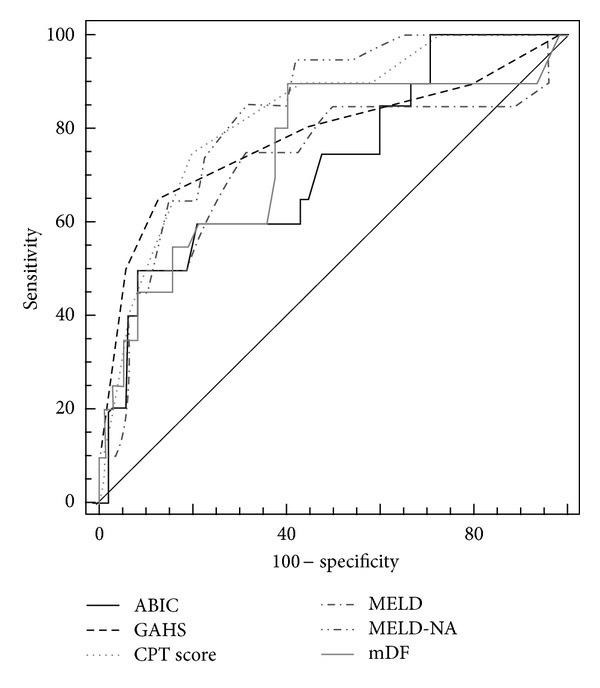
Receiver operating characteristic (ROC) curves for the studied scoring systems as predictors of 90-day mortality. For ABIC: AUC, 0.72; 95% CI 0.63–0.80. For CPT: AUC, 0.83; 95% CI 0.75–0.89. For GAHS: AUC, 0.78; 95% CI 0.69–0.85. For mDF: AUC, 0.75, 95% CI 0.66–0.82. For MELD: AUC, 0.72; 95% CI 0.63–0.80. For MELD-Na: AUC, 0.83; 95% CI 0.75–0.89.

These results are provided in Tables 5 and 6.  Figure 1 illustrates the comparison of AUCs for all models.

## 5. Discussion

Alcoholic hepatitis (AH) is common, but its pathogenesis, predictors of survival, and therapy remain debated [15]. Effective therapeutic support for pts with AH is dependent on a proper assessment of the disease severity and estimation of the risk of death as early in the course of the disease as possible. Several studies (mostly retrospective analyses) have been already performed to select predictors of mortality in pts with cirrhosis [5, 6, 16].

Since majority of them included pts with ALD as a subgroup of a larger studied cohort, conclusions that were drawn refer to a heterogeneous group of pts [17–19].

Martinez et al. [20] showed that survival of pts with type 1 hepatorenal syndrome associated with cirrhosis varied with liver disease etiology. Consequently, we designed the prospective study to investigate whether previous results can be extended for the homogeneous group of pts with AH. The factors we selected based on admission variables were similar to those reported by other researchers (e.g., bilirubin, serum albumin, INR, serum sodium level, mDF, CPT, MELD, MELD-Na, ascites, encephalopathy, and the CS nonresponse) [5, 18, 21]. Our important new finding is that AP > 1.5 ULN was revealed as an independent predictor of survival, after adjusting for all six risk scores and the CS nonresponse.

This variable has not been mentioned in previous reports. The second cholestatic enzyme-GGT was not associated with the outcome in our study (Table 1). Recently, Spahr et al. [22] have identified marked intraparenchymal cholestasis as an independent predictor of poor short-term outcome in their group of pts with histologically proven AH. Unexpectedly, only bilirubin and not AP level was related to 3-month mortality in those pts. In the current study both the previously mentioned laboratory variables were associated with poor short-term outcome as shown at the univariate analysis (bilirubin OR 1.11; 95% CI 1.04–1.18; *P* = 0.001 and AP > 1.5 ULN OR 3.85; 95% CI 1.31–11.32; *P* = 0.01). Furthermore, AP > 1.5 ULN was confirmed as an independent predictor of the poor 90-day survival.

We observed no significant difference in AP levels between CS responders and nonresponders (*P* = 0.87). However, results obtained in our study indicate that AP has an impact on the 90-day survival in AH. Two of 21 survivors treated with CS, who did not respond to the therapy, had lower AP levels in comparison with 11 of 20 nonsurvivors, CS nonresponders (156.5 ± 38.89 versus 220.7 ± 150.6, *P* = 0.58). The difference was not significant probably due to a small number of patients in the studied subgroups. On the other hand, 9 of 20 nonsurvivors, who responded to CS treatment, tended to have higher AP levels compared with 19 of 21 survivors and CS responders (251.9 ± 111.4 versus 186.4 ± 120.3, *P* = 0.06). Future studies are required to confirm our observations.

We have confirmed that mDF ≥ 32 predicted the poor outcome (it was present in 12 of 21 nonsurvivors versus 15 of 95 survivors, *P* = 0.0004) and was associated with AH complications. HE occurred in 15 of 27 pts with mDF ≥ 32 in comparison with 14 of 89 pts with mDF < 32, *P* = 0.0002. So, the subgroup of AH pts with mDF ≥ 32 requires particularly careful monitoring and intensive treatment. Unless contraindicated, CS is currently the best treatment option for them. However, previously published data indicate that about 40 percent of pts do not respond to CS [23] and so far no alternative medications have been found effective to cure AH. The rate of CS nonresponse in our study was 29.3 percent.

Since pts with mild forms of AH should not be treated with CS (not recommended for pts with mDF below 32 and/or without the presence of HE), the subgroup was treated with oral nutritional supplements (multivitamins and minerals, including folate and thiamine, and adequate protein intake, i.e., Nutridrink Protein Nutricia). In addition, salt restriction was applied after ascites had been confirmed.

The problem is that there is still no Food and Drug Administration-approved and/or widely accepted pharmacological therapy for any stage of ALD.

We are not able to answer what treatment options are superior in the subset of alcoholics with high AP serum level, because we have not investigated this issue. Future studies evaluating the impact of CS, as well as bile acids including ursodeoxycholic acid (UDCA), in pts with cholestatic type of AH (high AP serum level) are particularly needed. Preliminary data from a small clinical trial of UDCA treatment in AH pts have been already published and indicated a significant improvement in liver function tests [24].

On the other hand, Pelletier et al. [25] reported that UDCA administered at the dose recommended in primary biliary cirrhosis has no beneficial effect on the 6-month survival of patients with severe alcohol-induced cirrhosis. Although an inappropriate dosage of UDCA cannot be excluded as an explanation for the lack of therapeutic benefit.

So, the question if there is any difference in the therapeutic strategies between pts with high and low levels of AP should be further investigated.

The CPT, MELD, and MELD-Na score has been validated as independent predictors of survival in candidates for liver transplantation. Results from different trials have been contradictory. Some reports have demonstrated a statistical superiority of MELD over the CTP score [8, 18, 21, 26, 27], whereas others showed no statistical differences [18, 28, 29]. In our study, other scoring models, except for CPT, did not have an independent impact on the 90-day survival in AH.

Further studies were performed in order to improve the predictive ability of MELD score by adding clinical variables (hepatic encephalopathy, ascites) or laboratory parameters (sodium) [18, 30, 31]. As a result, the MELD-Na score was created being the most promising [9, 32–34]. Although significant at univariate analysis, low sodium level did not have an independent impact on 90-day outcome in our AH cohort after adjusted for AP > 1.5 ULN and the CS nonresponse.

Since Angermayr et al. [35] showed that etiology of liver cirrhosis may have a significant impact on survival predicted by MELD and that pts with identical scores, but different etiologies differed in survival rates, we aimed to assess accuracy of the scoring models in homogeneous group of pts with AH.

The analysis of AUCs in our study showed that all of the scores have proven good prognostic capabilities for the 3-month mortality, and hence, have similar utility in clinical practice. Our results are in line with the recent study by Sandahl et al. [36] who compared similar set of scoring systems (MELD, MELD-Na, GAHS, and ABIC) in Danish population and reported no statistically significant differences in the models' performances.

Although we showed that all models had an overall high accuracy, mDF ≥ 32 was less sensitive and ABIC presented the highest specificity in comparison with the other ones. Unexpectedly, the traditional CPT had quite good performance with the highest AUC (together with newer MELD-Na). Furthermore, incorporation of AP > 1.5 ULN and the CS nonresponse in the same statistical model together with CPT increased its accuracy (0.91 versus 0.83); nonetheless, the difference was not statistically important. However, the significance was found when AUC of the complex CPT statistical model was compared with AUCs of ABIC and MELD (*P* = 0.015 and  *P* = 0.027, resp.) (Tables 4 and 5).

The exact cut points of the CPT, MELD, and MELD-Na that best predict mortality are not clearly established. In our study, the cutoff values of 10 for CPT, 18 for MELD, and 21 for MELD-Na were close to those reported in previous trials by Dunn et al. [6], Srikureja et al. [8], and Sheth et al. [37] but lower than those reported by Vaa et al. [9] and Soultati et al. [38]. Noteworthy, Somsouk et al. [39] indicate that, although MELD and MELD-Na captures short-term mortality risk well especially at high values, ascites may represent an important marker of liver decompensation not captured by low MELD and MELD-Na scores. They showed that liver transplant waitlist mortality was higher in pts with moderate ascites and low MELD values (<21). The data indicate that, apart from the precise cut points of scoring models, other factors should prompt clinicians to consider strategies to expand access to liver transplantation.

Our study has some limitations. It was a single-center trial. The results of nonsurvivors should be interpreted with caution due to the relatively small size of the subgroup. The majority of our pts did not have biopsy proven AH. Liver biopsy, the “gold standard” for diagnosis of AH, is frequently not feasible in the clinical setting due to the presence of ascites or coagulopathy. Few liver centers in Poland, and to our knowledge in Europe, are able to provide a transjugular procedure as an option. Nevertheless, clinical criteria for the diagnosis of AH have been widely used for recruitment of subjects into clinical trials [5, 16, 40]. Furthermore, the recent review of the randomized controlled trials performed by Hamid and Forrest shows that the accuracy of a clinical diagnosis of AH based on a minimum level of bilirubin ranging from 80 to 100 *μ*mol/L was about 96% [13]. 

Further studies are required to ascertain our results in the future.

## 6. Conclusions

Our study demonstrates that the CPT score, AP > 1.5 ULN, and the CS nonresponse were identified as independent predictors of the poor 90-day survival in AH. We hypothesize that the addition of AP > 1.5 ULN to existing disease severity scores may improve their prognostic capabilities at admission. Accuracy of all studied scoring systems was comparable in predicting 3-month outcome in AH. The most compelling argument for poor prognosis was ABIC greater than 8.46 and against it MELD-Na equal to or lower than 21. Incorporation of AP > 1.5 ULN and the CS nonresponse into the statistical model together with CPT increased its accuracy and was better than individual ABIC and MELD performances.

## Figures and Tables

**Table 1:** Baseline characteristics of the study population^#^.

	Total group (*n* = 116)		Survivors (*n* = 95)		Nonsurvivors (*n* = 21)	
	Mean	SD	Mean	SD	Mean	SD
Age/years	49.35	10.66	49.32	10.66	49.50	10.95
Tbil*** mg/dL	9.13	6.78	8.04	5.37	14.31	9.95
Alb*** g/dL	3.02	0.54	3.14	0.49	2.49	0.44
INR***	1.47	0.41	1.41	0.34	1.80	0.53
PT*** s	16.61	4.29	15.86	3.63	20.17	5.37
Crea mg/dL	1.10	0.94	1.07	1.02	1.21	0.38
Na** mEq/L	136.25	5.01	136.85	4.62	133.40	5.88
ALT U/L	73.30	64.63	74.43	69.06	67.95	37.82
AST U/L	155.36	105.06	155.76	112.14	153.50	63.33
AP** U/L	179.68	102.39	168.54	92.79	235.35	130.48
GGT U/L	927.00	1279.59	1007.80	1374.00	494.47	307.53
WBC* ×10^3^/*μ*L	10.17	6.39	9.58	5.96	12.93	7.70
Hgb g/dL	11.50	1.87	11.57	1.98	11.17	1.25
RBC ×10^6^/*μ*L	3.46	0.65	3.50	0.66	3.25	0.56
PLT ×10^3^/*μ*L	147.28	85.39	148.03	87.70	143.70	75.38
CRP* mg/L	39.20	52.16	34.32	50.31	61.42	55.94
Gender M/F	72/44		61/34		11/10	
Ascites*** (+/−)	74/42		53/42		21/0	
HE** (+/−)	29/87		18/77		11/10	
CS** response (+/−)	29/12		20/2		9/10	

***P < 0.001; **P < 0.01; *P < 0.05 variables significantly different in nonsurvivors versus survivors; ^#^Alb: albumin (NR 3.2–4.8); ALT: alanine aminotransferase (normal range (NR) <31); AP: alkaline phosphatase (NR 45–129); AST: aspartate aminotransferase (NR <34); CS: corticosteroids; Crea: creatinine (NR 0.5–1.1); CRP: C-reactive protein (NR 0.0–5.0); F: female; GGT: gamma-glutamyl transpeptidase (NR <50.0); HE: hepatic encephalopathy; Hgb: hemoglobin (NR 14.0–18.0); INR: International Normalized Ratio (NR 0.8–1.2); M: male; Na: sodium (NR 136–145); PLT: platelets (NR 130–400); PT: prothrombin time (NR 10.4–13.0); q25: first quartile; q75: third quartile, RBC: red blood cells (NR 4.5–6.1); SD: standard deviation, Tbil: total bilirubin (NR 0.3–1.2); WBC: white blood cells (NR 4.8–10.8).

**Table 2:** Baseline scoring systems values of the study population (*n* = 116)^#^.

	Survivors (*n* = 95)				Nonsurvivors (*n* = 21)			
	Mean	SD	Median	q25–q75	Mean	SD	Median	q25–q75
ABIC**	7.02	1.15	7.01	6.23–7.78	7.97	1.17	8.15	6.96–8.60
GAHS***	7.44	1.16	7.00	7.00-8.00	9.15	1.78	9.50	8.00–10.00
MELD***	17.74	4.90	16.00	15.00–19.75	22.80	7.28	22.50	18.00–28.00
MELD-Na***	20.40	7.15	19.00	17.00–22.00	27.70	7.25	26.00	22.50–31.00
CPT***	9.00	1.69	9.00	7.00–10.00	11.05	1.36	11.00	10.50–12.00
mDF***	21.11	17.66	15.00	10.03–27.10	46.78	34.77	32.00	20.00–68.71

***P < 0.001; **P < 0.01.

^#^ABIC: Age, Bilirubin, INR, Creatinine score; CPT: Child-Pugh-Turcotte score; GAHS: Glasgow Alcoholic Hepatitis Score; mDF: modified Maddrey's discriminant function; MELD: Model for End-Stage Liver Disease; q25: first quartile; q75: third quartile.

**Table 3:** Predictors of 90-day mortality (univariate logistic regression)^#^.

Variable	Odds ratio	95% CI	*P* value
ABIC	2.01	1.28 to 3.16	0.0024
GAHS	2.28	1.55 to 3.36	<0.0001
MELD	1.15	1.06 to 1.25	0.0008
MELD-Na	1.11	1.04 to 1.19	0.0016
CPT	2.28	1.52 to 3.42	0.0001
mDF	1.04	1.02 to 1.06	0.0002
Tbil	1.11	1.04 to 1.18	0.0010
Albumin	0.03	0.005 to 0.15	<0.0001
INR	8.87	2.52 to 31.25	0.0007
Na ≤ 135 mEq/L	3.82	1.35 to 10.84	0.0117
AP > 1.5 ULN	3.85	1.31 to 11.32	0.0142
WBC	1.08	1.00 to 1.15	0.0386
HE	4.28	1.55 to 11.81	0.0050
CS nonresponse	11.61	2.11 to 63.73	0.0048

^#^CI: confidence interval.

**Table 4:** Independent predictors of 90-day mortality (multivariate logistic regression)^#^.

Variable	Adjusted odds ratio	95% CI	*P* value	AUC (95% CI)
CPT	1.88	1.07–3.32	0.029	0.91 (0.76–0.98)
AP > 1.5 ULN	11.55	1.28–103.91	0.029	
CS nonresponse	24.77	2.14–286.52	0.010	

^#^AP: alkaline phosphatase; AUC: area under the curve; CI: confidence interval; CPT: Child-Pugh-Turcotte score; CS: corticosteroids.

**Table 5:** Comparison of the areas under the curves (AUCs) for the studied models.

Scoring system	AUC	SE^a^	95% CI^b^
ABIC*	0.72	0.06	0.63–0.80
GAHS	0.78	0.07	0.69–0.85
MELD*	0.72	0.07	0.63–0.80
MELD-Na	0.83	0.04	0.75–0.89
CPT	0.83	0.05	0.75–0.89
mDF	0.75	0.07	0.66–0.82

^a^SE: standard error, DeLong; ^b^binomial exact; *P < 0.05 in comparison with AUC of the complex CPT model (CPT + AP > 1.5 ULN + CS nonresponse).

**Table 6:** The best threshold values of the studied models for predicting 90-day outcome.

Scoring system	Criterion	Sensitivity (95% CI)	Specificity (95% CI)	+LR^#^	−LR
ABIC	>8.46	50.00 (27.2–72.8)	91.58 (84.1–96.3)	5.94	0.55
GASH	>8	65.00 (40.8–84.6)	87.37 (79.0–93.3)	5.15	0.40
MELD	>18	75.00 (50.9–91.3)	68.42 (58.1–77.6)	2.37	0.37
MELD-Na	>21	85.00 (62.1–96.8)	68.42 (58.1–77.6)	2.69	0.22
CPT	>10	75.00 (50.9–91.3)	80.00 (70.5–87.5)	3.75	0.31
mDF	>32	45.00 (23.1–68.5)	84.21 (75.3–90.9)	2.85	0.65

^#^LR: likelihood ratio.
